# Marine subsidy promotes spatial and dietary niche variation in an omnivore, the Keen’s mouse (*Peromyscus keeni*)

**DOI:** 10.1002/ece3.8225

**Published:** 2021-12-06

**Authors:** Katie H. Davidson, Brian M. Starzomski, Rana El‐Sabaawi, Morgan D. Hocking, John D. Reynolds, Sara B. Wickham, Chris T. Darimont

**Affiliations:** ^1^ Department of Geography University of Victoria Victoria British Columbia Canada; ^2^ Hakai Institute Heriot Bay British Columbia Canada; ^3^ School of Environmental Studies University of Victoria Victoria British Columbia Canada; ^4^ Department of Biology University of Victoria Victoria British Columbia Canada; ^5^ Ecofish Research Ltd. Victoria British Columbia Canada; ^6^ Department of Biological Sciences Simon Fraser University Burnaby British Columbia Canada; ^7^ Raincoast Conservation Foundation Sidney British Columbia Canada; ^8^ Present address: School of Environment, Resources and Sustainability University of Waterloo Waterloo Ontario Canada

**Keywords:** allochthonous, individual niche, islands, Keen's mouse, *Peromyscus keeni*, resource pulses, stable isotopes, wrack

## Abstract

Marine‐derived resource subsidies can generate intrapopulation variation in the behaviors and diets of terrestrial consumers. How omnivores respond, given their multiple trophic interactions, is not well understood. We sampled mice (*Peromyscus keeni*) and their food sources at five sites on three islands of the Central Coast of British Columbia, Canada, to test predictions regarding variation in the spatial behavior and consumption of marine‐subsidized foods among individuals. About 50% of detections (*n* = 27 recaptures) occurred at traps closest to shoreline (25 m), with capture frequencies declining significantly inland (up to 200 m). Stable isotope signatures (*δ* ^13^C and *δ* ^15^N), particularly *δ* ^15^N, in plant foods, forest arthropod prey, and mouse feces were significantly enriched near shorelines compared with inland, while *δ* ^13^C patterns were more variable. Bayesian isotope mixing models applied to isotope values in mouse hair indicated that over one‐third (35–37%) of diet was comprised of beach‐dwelling arthropods, a marine‐derived food source. Males were more abundant near the shoreline than females and consumed more marine‐derived prey, regardless of reproductive status or availability of other food sources. Our results identify how multiple pathways of marine nutrient transfer can subsidize terrestrial omnivores and how subsets of recipient populations can show variation in spatial and dietary response.

## Graphical abstract

Marine‐derived resource subsidies can generate intrapopulation variation in the behaviors and diets of terrestrial consumers. Isotope mixing models indicated that over one‐third (35–37%) of mouse diets were composed of marine‐derived foods, and males consumed more marine‐derived prey than females, regardless of reproductive status or availability of other food sources. Our results indicate multiple pathways for marine subsidy and corresponding variation within populations in spatial patterns and diets.

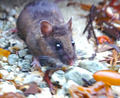

## 1 INTRODUCTION

Resource subsidies (also called “allochthonous resources”), which originate from adjacent habitats, occur globally and link ecosystems through the flow of nutrients and/or organisms. A significant body of literature has documented their varied effects on recipient consumer populations (reviewed in Marczak et al., 2007; Polis et al., 1997). Resource subsidies can, for example, stabilize food webs by diversifying the resource portfolios of consumers (Huxel & McCann, 1998; Leroux & Loreau, 2008; Polis & Strong, 1996; Service et al., 2018), but the trophic position at which the subsidy enters the recipient food web (Leroux & Loreau, 2008; Marczak et al., 2007; Polis et al., 1997), the traits and interaction strengths of recipient consumers (Gratton & Denno, 2003; Huxel et al., 2002; Kopp & Allen, 2021; Schilke et al., 2020), and recipient habitat heterogeneity (Marczak et al., 2007; Polis & Strong, 1996) all can play a role in determining the specific outcomes of resource subsidies. For example, in systems where resource subsidies initiate trophic cascades, the behavior of generalist consumers has been hypothesized to affect the magnitude of the cascade (Leroux & Loreau, 2008).

Omnivores are an example of a generalist consumer, and their response to resource subsidies, along with the resulting impact on food web components, may be varied. They may respond rapidly to resource subsidies given their dietary plasticity, or may maintain a diet that is diversified across multiple food web channels (e.g., plant and animal matter; Polis & Strong, 1996; Vadeboncoeur et al., 2005). Furthermore, the trophic position of both the omnivorous consumer and the subsidy source within the recipient food web likely impacts the effects of subsidy. Lower trophic level omnivores may experience both “top‐down” regulation from predators and “bottom‐up” regulation, especially from resource subsidies from primary producers, thus altering the impacts of resource subsidies (Leroux & Loreau, 2008). However, low‐trophic level consumers, especially omnivores, are rarely the focal consumer in subsidy research.

Within omnivore populations, it is not clear whether individuals or groups within the population respond uniformly to marine subsidies. There is evidence that within consumer guilds and populations, differences in foraging strategies and dietary plasticity (Schilke et al., 2020), life stages (e.g., reproductive versus juvenile; Ben‐David et al., 2004; Hailey et al., 2001; Wolf & Batzli, 2002), and individual traits (e.g., body size and dispersal ability; Gratton & Denno, 2003; Kopp & Allen, 2021) can impact which individuals or groups exploit subsidies. These finer‐scale nuances of subsidy effects are less studied, but are crucial in understanding whether such patterns “scale up” across ecosystems. Studies thus far have mostly focused on terrestrial‐aquatic (freshwater or lake) arthropod systems where linkages are strong; coastal ecotones between marine and terrestrial ecosystems may exhibit similar linkages, but are thought to be difficult to characterize (Leroux & Loreau, 2008).

Wrack (shore‐cast algae) is a marine subsidy linking marine and terrestrial environments across coastal ecotones, and might fuel food webs involving terrestrial omnivores. Vast stretches of coastline receive wrack (shore‐cast macroalgae), a consistent and ubiquitous source of marine subsidy (Barreiro et al., 2011; Orr et al., 2005; Wickham et al., 2020). Its presence provides an opportunity to study effects of a “press” subsidy, delivered throughout the year, as opposed to a more common “pulse” subsidy, for example, the annual return of adult Pacific salmon (*Onchorynchus* spp.) to spawn (Reimchen, 2000; Walters et al., 2018; Yang et al., 2010). It is indirectly incorporated into terrestrial food webs by enriching soil and vegetation with marine forms of nitrogen (Del Vecchio et al., 2013; Piovia‐Scott et al., 2013; Spiller et al., 2010; Williams & Feagin, 2010) or through direct consumption of wrack‐associated primary consumers such as amphipods and weevils, which increase in abundance with wrack biomass (Barrett et al., 2005; Dugan et al., 2003; Stapp & Polis, 2003a). Thus, different pathways of subsidy can directly and indirectly impact the diets of terrestrial consumers occupying shorelines and nearby habitats, particularly on small oceanic islands with high perimeter‐to‐area ratios (Obrist et al., 2020; Polis et al., 1997). How abiotic and biotic factors might interact on these islands to influence foraging and associated spatial behaviors of consumers remains less examined.

Small mammals, particularly mice, are omnivores that occupy a broad dietary niche, making them good model organisms to understand how resource subsidy might influence intrapopulation variation in dietary and spatial niches. In fully terrestrial systems with no marine influence, mice (*Peromyscus* spp.) exhibit seasonal changes in dietary niche to exploit high‐quality, pulsed resource subsidies as anticipated under optimal foraging theory (Stephens et al., 2019). On islands in dry regions, where terrestrial productivity is low, wrack provides an important, temporally consistent nutrient subsidy to coastal deer mice (*Peromyscus maniculatus*) and their food sources (Stapp & Polis, 2003a, 2003b). In the Pacific Northwest, where terrestrial productivity is relatively higher, Keen's mice (*Peromyscus keeni*) exploit wrack‐derived marine resources, but detailed studies are lacking. Early work documented more Keen's mice along shorelines compared with forest interiors, a pattern long ago hypothesized to be driven by consumption of amphipods associated with wrack on beaches (McCabe & McTaggart‐Cowan, 1945, Thomas, 1971, but see Marinelli & Millar, 1989). This early inference was based largely on observations of mice (and mouse tracks) on beaches (McCabe & McTaggart‐Cowan, 1945), as well as evidence of amphipod exoskeletons in mouse stomachs (Thomas, 1971). Larger mice, and a higher frequency of pregnant females near shorelines also suggested that a high‐protein food source, presumably composed of intertidal prey, was available to those individuals and may be targeted during energetically demanding life stages (Marinelli & Millar, 1989). These studies, however, are limited in number and in the insight possible from the historical methods applied. Using a suite of new approaches, we were interested in understanding how spatial and dietary behavior might vary within populations, and how such variation might correspond to demographic (i.e., sex‐ and reproductive‐class) or environmental (i.e., food availability) factors.

To address this overarching objective, we examined the foraging ecology, pathways of marine subsidy, and spatial and dietary niche variation among individuals in populations of omnivorous Keen's mice (*Peromyscus keeni*) along island shorelines in Haíɫzaqv (Heiltsuk) and Wuikinuxv First Nations Territories on the Central Coast of British Columbia, Canada. We sampled sites at extreme ends of a known marine subsidy (wrack) gradient in the region (Wickham et al., 2020) to capture the range of marine enrichment in these coastal food webs. We obtained demographic and biometric information, along with tissue samples from Keen's mice and their food items for stable isotope (carbon [*δ* ^13^C] and nitrogen [*δ* ^15^N]) analysis.

Based on the theory and empirical patterns discussed above, we made several predictions. First, we hypothesized that a direct subsidy pathway from wrack to mice exists via the consumption of marine‐derived, “beach‐dwelling” arthropods (e.g., amphipods, Family Talitridae) associated with wrack patches, as opposed to a solely indirect pathway through which marine nutrients are incorporated indirectly, and via forest food sources (e.g., plant or forest arthropods) that are marine‐subsidized. Second, if these beach‐dwelling arthropods comprise important dietary items, we hypothesized that mice would occupy shorelines more frequently than forest interiors. Finally, we used environmental (prey abundance) and individual (sex, breeding status) variables to test hypotheses about dietary variation among individuals. Specifically, whereas low terrestrial productivity has been hypothesized to increase consumption of marine‐derived foods by small mammals (Stapp & Polis, 2003a, 2003b), we hypothesized that terrestrial productivity (both vegetation and invertebrate food abundance) would be less important than the abundance of marine foods in our study area where terrestrial productivity is relatively higher. Moreover, we predicted that reproductive females would be captured more frequently than males near the beach and consume more marine‐derived foods than males (Marinelli & Millar, 1989).

## 2 METHODS

### 2.1 Study area and field sampling

**FIGURE 1:**
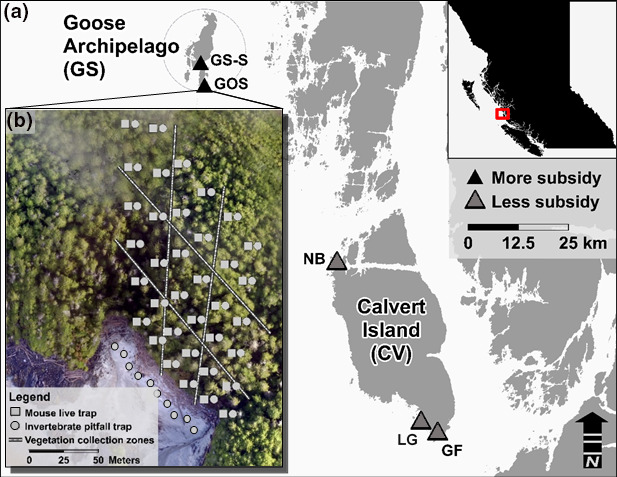
(a) Study sites in Haíɫzaqv (Heiltsuk) and Wuikinuxv Territories on the Central Coast of British Columbia, Canada. Goose Island South (GS‐S) and Gosling Island (GOS) in the Goose Archipelago (GS) represent sites with higher subsidy than North Beach (NB), Grief Bay (GF), and Little Grief (LG) on Calvert Island (CV). (b) An example sampling grid at GOS showing approximate vegetation collection zones, mouse live traps (squares), and arthropod pitfall traps (circles) in the forest and littoral zone

We conducted our sampling adjacent to sandy beaches on Calvert Island (North Beach, Grief Bay and Little Grief Bay), Gosling, and Goose Islands (within the Goose Archipelago) on the Central Coast of British Columbia, Canada (Figure 1). Previous work has documented more wrack deposition on Goose Archipelago beaches than those on Calvert Island, although wrack species composition was significantly different between the two regions (Wickham et al., 2020), suggesting that the Goose Archipelago receives more marine nutrients than Calvert Island.

Sampling occurred in late May (Grief Bay and Little Grief), mid‐July (North Beach), late August (South Goose), and early September (Gosling) of 2016, except for fecal sampling, which occurred late August 2015 on Gosling Island. At each site, we set a grid of trap stations extending from the beach into the forest (Stapp & Polis, 2003a, 2003b). Trapping grids were 4–6 trap lines wide, and 6–9 trap stations deep, depending on the site. Trap lines were spaced 15 m apart, with each trap station spaced at 25‐m intervals along the trap line extending away from the beach (125–200 m inland, depending on site) (Figure 1).

Each trap station included one Sherman small folding aluminum live mouse trap (~0.6 mm aluminum, 7.6 × 9.5 × 30.5 cm, product code SFAL, H. B. Sherman Traps Inc., Tallahassee, FL) and one arthropod pitfall trap (500 ml plastic yogurt container, ~11‐cm diameter opening, ~8‐cm depth). Sherman traps were baited with a small amount (~0.5 g) of peanut butter and set over 1–5 nights (but checked daily) at each site depending on weather and logistical constraints (see Appendix for more trapping details). We obtained body condition metrics (weights and hindfoot length), demographic traits such as sex, breeding status, and age (adult or subadult), and hair samples from all mice captured. At two sites (Grief Bay and Little Grief Bay), individual mice were also marked with colored bands around the base of their tail and on their ventral surface (using Stoelting^®^ Animal Markers) to provide spatial data for movement analyses (below).

Arthropod pitfall traps in the forest were set for 3–6 nights and were set at all sites except Little Grief Bay. We also placed pitfall traps 1–2 m above the highest recent tide line to sample beach‐dwelling arthropods, setting them for 1 night. Arthropods were identified to the lowest taxonomic level possible (typically family or finer when possible) and grouped as carnivores, herbivores, and detritivores for biomass and stable isotope analysis (Appendix Table A1). Given our objective was to obtain species that would be isotopically representative of invertebrate prey consumed by mice, and to represent relative biomass between regions and across distance intervals, we used pitfall traps (Corti et al., 2013; Sabu & Shiju, 2010; Standen, 2000), acknowledging their potential to overestimate ground‐dwelling, fast‐moving arthropods (Andersen, 1995; Standen, 2000).

Due to the patchy distribution of fruit‐bearing shrubs at our study locations, we collected berries and fruits at zones at the front (i.e., beach; 0–25 m), middle (50–75 m), and back (100–200 m) of the trapping grids for stable isotope analysis (Figure 1; see Appendix Tables A3, A4 and A3, A4 for species). We used isotopic signatures of berries and fruits as a proxy for overall plant isotopic signature, but variable fractionation across plant anatomy (i.e., vegetative or reproductive tissue) may result in differential *δ* ^13^C signatures (Brüggemann et al., 2011), which could be passed on to herbivorous consumers depending on foraging habits. Vegetation samples were only collected from North Beach, South Goose, and Gosling Island. We avoided sites of past human occupation (i.e., seasonal village or habitation sites), which have been shown to differ in soil and plant nutrient content compared with more historically diffusely inhabited sites (Fisher et al., 2019; Trant et al., 2016).

We used track plates to passively collect fecal samples from mice at two sites on Gosling Island. Track plates were organized in a similar grid as above, although grids were only three trap lines wide. Fecal samples were obtained from track plates after 2–4 nights and then pooled (across each trap night) into one fecal sample for stable isotope analysis. Track plates were baited with a small amount (~0.5 g) of peanut butter on the inner roof. Although baiting may potentially influence isotopic signatures in fecal samples, comparisons among distance intervals from the beach remain possible.

Details on trap design (mouse and arthropod), mouse biometric measurements, and sample storage and processing are in the Appendix.

### 2.2 Spatial subsidy patterns in mice and food items

We calculated the number of adult male and female mice captured, accounting for trapping effort, per 100 trap nights (Nelson & Clark, 1973) at four distance intervals (0–25, 50–75, 100–125, and 150–200 m). Mice were assigned to intervals based on the location of first capture and pooled across trap lines for each distance interval by site, yielding a catch per unit effort (CPUE) index (Stapp & Polis, 2003a). Using CPUE, we calculated the proportion of captures for each distance interval at each site (*n* = 5) and compared them using a two‐way ANOVA (for sex and distance interval) and Tukey's post hoc tests. We ensured that assumptions of normality and equal variance were met by visually examining residual and quantile–quantile plots before proceeding with parametric tests. We used the *Anova* function from the *car* package (Fox & Weisberg, 2019) to account for potential effects of an unbalanced two‐way ANOVA design. Adult mouse body mass was compared across distance intervals for males and females separately using non‐parametric Kruskal–Wallis tests. We examined movement around the trapping grid for individuals with a minimum of three recapture events.

We also estimated forest arthropod biomass (dry weight) per pitfall trap, accounting for sampling effort (see Appendix Methods for sample processing details). Biomass data were base‐10 log‐transformed and analyzed among distance intervals using a one‐way ANOVA. We did not compare changes in biomass among trophic groups due to potential trophic bias arising from pitfall traps (Andersen, 1995; Sabu & Shiju, 2010; Standen, 2000). We present biomass data as overall mean (± standard error) per distance interval (0–25, 50–75, 100–125, 150–200 m).

Finally, we examined spatial patterns in *δ* ^13^C and *δ* ^15^N signatures in mouse feces, hair, and food items from the beach inland at the same four distance intervals as above (0–25, 50–75, 100–125, and 150–200 m). For food sources (fruits/berries and arthropods), we selected the most common taxa as representatives for each trophic level: Salal (*Gaultheria shallon*) represented fruits/berries, weevils (*Steremnius carinatus*) represented herbivorous forest arthropods, and Carabid ground beetles (*Scaphinotus angusticollis*, *Pterostichus lama*, *Zactous matthewsii*, and *Cychrus tuberculatus*) represented carnivorous forest arthropods. Mouse hair and feces were compared across distance intervals using one‐way ANOVA and Tukey's post hoc tests, whereas food items were each compared across distance intervals using Kruskal–Wallis and Dunn's post hoc tests (Dinno, 2017). Statistical analyses and plots are presented as raw isotopic values without trophic fractionation values applied.

We did not correct for lipid content in any tissues used in isotope mixing models to preserve assumptions of isotope mass balance, particularly when high‐lipid prey (e.g., some arthropods and plant material) are consumed whole (Arostegui et al., 2019). However, due to high and variable C:N ratios in mouse feces and forest arthropods, and recommendations in Post et al. (2007), we used a linear model (equation 6 in Post et al., 2007) to lipid‐correct *δ* ^13^C signatures in these two tissues to examine spatial patterns in *δ* ^13^C (C:N 8.8–14.3 and 4.1–8.4 in feces and arthropods, respectively).

### 2.3 Stable isotope diet modeling

We used mouse hair and food signatures within an isotope mixing model, MixSIAR, to estimate the relative contribution of food items to individual mouse diets (Stock et al., 2018). These dietary estimates provide an opportunity to assess the relative roles of direct versus indirect subsidy to mice. Specifically, estimates identify what proportion of food comes directly from the ocean in the form of beach‐dwelling arthropods (one of the food sources, see below) and what proportion is derived from terrestrial sources (see below) that themselves may be enriched by marine resources (i.e., indirect paths of subsidy to mice). We modeled diets for 73 individual mice, including those from Little Grief. Although we did not have food source data from Little Grief, we assumed the food source samples from the other two Calvert Island sites (Grief Bay and North Beach) were isotopically representative of what mice at Little Grief would consume, given the geographic proximity (all sites are located on Calvert Island; see Figure 1) and habitat similarity.

Due to significant differences in the *δ* ^13^C and *δ* ^15^N signatures of food items between the Goose Archipelago and Calvert Island (Appendix Table A2), we created separate mixing models to estimate individual mouse diets from these two relatively distant regions. Although arthropods were initially recorded as trophic groups (detritivore, herbivore, and carnivore) for biomass estimates, we used mean values across trophic groups to create three food sources that isotopically represented the primary items in mouse diets: vegetation, forest arthropods, and beach arthropods (Drever et al., 2000; Marinelli & Millar, 1989; Sullivan, 1977; Thomas, 1971) (Appendix Tables A3, A4 and A3, A4). We had three reasons for this approach. First, variation among locations (forest vs. beach) and sources (plant vs. arthropod) was greater than variation between trophic levels (e.g., herbivorous forest arthropod vs. carnivorous forest arthropod). Second, early model iterations included five food sources: vegetation, herbivorous forest arthropods, carnivorous forest arthropods, herbivorous beach arthropods, and carnivorous beach arthropods. The estimate of marine‐derived foods in diet did not change when we simplified the model to three food sources, and model convergence diagnostics became more reliable. Finally, we simplified the model to three food sources to align with recent recommendations in the literature (Phillips et al., 2014).

We used the mean (± standard deviation) *δ* ^13^C and *δ* ^15^N signatures for each of the three food groups, and applied trophic fractionation values of +3.3‰ for *δ* ^15^N, and *δ* ^13^C fractionation values of +1‰ for arthropod tissue and +2‰ for plant tissue (Drever et al., 2000). MixSIAR models were run on “very long” (chain length = 1,000,000; burn = 500,000; thin = 500; chains = 3) to ensure Gelman–Rubin and Gweke tests produced acceptable diagnostic values (Stock & Semmens, 2016), with “individual” as a random effect for individual estimates (used in the GLMM below). We also ran MixSIAR models on “very long” for overall regional estimates provided in Table 1. We report all dietary proportions as medians with 95% credible intervals.

**TABLE 1:** Diet proportions (MixSIAR regional median posterior distribution values with 95% credible intervals) of each source food group in diets of Keen's mice from Calvert Island (*n* = 31) and the Goose Archipelago (*n* = 18)

Location	Calvert Island	Goose Archipelago
Berries and fruits77	31.9 (4.5–48.4)	11.6 (1.1–31.9)
Forest arthropods	30.7 (5.1–75.7)	53.3 (25.5–69.5)
Beach arthropods	37.0 (19.4–47.8)	35.4 (26.6–45.2)

### 2.4 Examining variation in consumption of marine‐derived prey

We used generalized linear mixed‐effects models (GLMMs) to investigate how individual (i.e., sex and breeding stage) and environmental factors might influence the relative quantity of marine‐derived foods in individual diets. We modeled these relationships for 44 individual mice; we did not include mice from the Little Grief site because data for environmental variables were unavailable.

For our analysis, we used the median values of the estimated posterior density distributions of the proportion of beach arthropods in individual diets from our MixSIAR model outputs (Service et al., 2018). The proportion of beach arthropods provides a comprehensive representation of the possible contribution of marine‐derived foods to individual diets because it considers all potential food sources simultaneously. We acknowledge, however, that there are two considerations when using isotopic mixing‐model outputs to evaluate resource use and availability. First, the posterior distributions often exhibit wide credible intervals of dietary proportions and contain inherent variation and error. Second, mixing‐model outputs are derived values that require several assumptions to calculate “true” representations of dietary contributions (reviewed by Phillips et al., 2014). To address the first concern, we repeated the GLMM process using the 5% and 95% values from the estimated poster density distributions of the proportion of beach arthropods in individual diets to examine consistency in model results. To address the second concern, we used the same modeling process and predictor variables described below to examine variation in *δ* ^13^C and *δ* ^15^N signatures in individual mouse hair, which represents the “raw” isotopic enrichment in each individual's diet.

We modeled our response variables against five predictor variables: beach arthropod biomass (site level), forest arthropod biomass (trap and site level), Normalized Differentiated Vegetation Index (trap‐ and site‐level NDVI), mouse sex, mouse reproductive status (reproductive or non‐reproductive), and an interaction between sex and reproductive status (Appendix Table A5).

NDVI represents forest vegetation productivity (see Appendix Methods for details). NDVI captures infrared wavelengths re‐emitted by leaves (typically of the upper canopy) during photosynthesis, yielding a proxy for photosynthetically active biomass and, consequently, productivity (ranging from −1.0 to 1.0, low to high photosynthetic activity, respectively). We assume that higher NDVI values indicate increased availability of vegetative food (but see discussion in Appendix Methods). Forest arthropod biomass and NDVI were analyzed at the site and trap level to account for the different scales at which these predictor variables might influence foraging by mice.

We centered and scaled continuous predictors (biomass and NDVI) by subtracting the sample mean and dividing by two times the sample standard deviation (Gelman, 2008; Appendix Table A5). We investigated potential collinearity of predictor variables using regression plots and Generalized Variance Inflation Factors (GVIF) (Fox & Weisberg, 2019). In all analyses, variables exhibited GVIF < 2, which were within broadly acceptable limits of collinearity (Chatterjee & Hadi, 2006; O’Brien, 2007, but see Graham (2003)). Variables with *r* > .6, however, were not considered in the same candidate models (Appendix Table A6). Pairwise correlations with *r* > .6 included forest arthropod biomass at the site and trap levels (*r* = .71); site‐level forest arthropod biomass and beach arthropod biomass (*r* = −.64); NDVI‐site and site‐level forest arthropod biomass (*r* = −.73); and NDVI‐trap and trap‐level forest arthropod biomass (*r* = −.70).

We created sets of candidate models using a combination of *a priori* biological knowledge and the above cutoff for correlated variables. For all of the above modeling exercises, we used the same set of candidate models and predictor variables (Appendix Table A6). Owing to small sample size, we limited models to include a maximum of three predictor variables in any given model, except when models included an interaction between “sex” and “breeding status” (sex*breeding status), in which case we allowed four. We assumed the “proportion of beach arthropods” response variables were beta‐distributed (logit link) and fit beta‐GLMMs using the *glmmADMB* package (Fournier et al., 2012; Skaug et al., 2016). We assumed the isotope response variables (*δ* ^13^C and *δ* ^15^N) were Gaussian‐distributed (identity link), and fit Gaussian linear mixed‐effects models (LMMs) using the *lme4* package (Bates et al., 2015). All models in each process were competed using Akaike's information criterion corrected for small sample sizes (AICc) (Burnham & Anderson, 2002) with the *AICcmodavg* package (Mazerolle, 2019). Models were ranked based on ΔAICc scores. We considered top models those that accounted for 95% of total model weight. We used the *MuMIn* package (Barton, 2019) to calculate full model‐averaged parameter estimates, and compared AICc weight ratios and parameter (± SE) estimates to assess the relative effects of predictor variables on the dietary response variables. We did not calculate relative variable importance (RVI) scores due to an unbalanced candidate model set, but instead considered evidence ratios ($w_{\text{top model}}$ divided by $w_{\text{subsequent models}}$, where *w* is the model weight). All statistical analyses and modeling were conducted using R in RStudio (R Core Team, 2019), and we assessed significance differences at α = 0.05. An overview of all variables, details and processing of data used in mixed‐effects models is included in Appendix Table A5.

## 3 RESULTS

### 3.1 Shoreline to forest patterns in mice and food items

**FIGURE 2:**
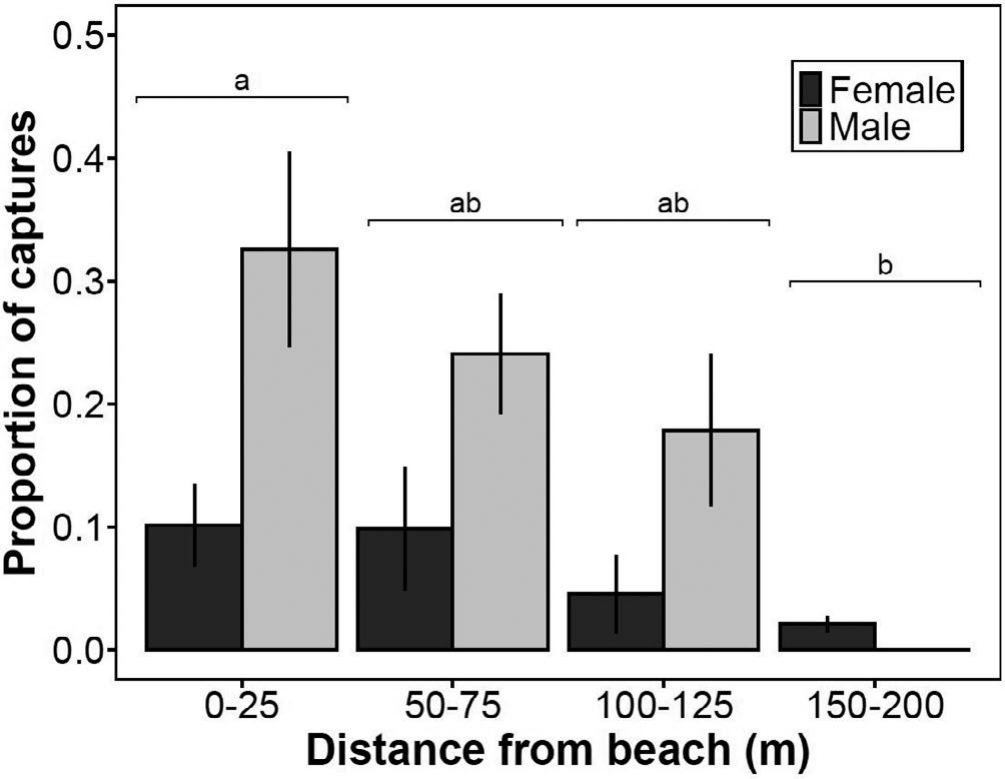
Average (± 1 *SE*) proportion of Keen's mouse (*P*. *keeni*) captures at each distance interval from the beach to the forest across all sites (excluding recaptures) for males and females. Based on CPUE per 100 trap nights across all five sites. No males were caught at 150–200 m. Letters indicate statistical comparisons among capture frequencies at different distance intervals (two‐way ANOVA; sex‐based comparison results not shown); different letters indicate statistical significance (Tukey's post hoc test, *p* < .05)

We captured 55 unique adult mice (*n* = 20 females, 35 males) across 5 sites. Averaged across sites, we caught 44% (± 9.4%) of the individuals within 25 m from shore. After correcting for effort (i.e., CPUE per 100 trap nights), we caught a significantly higher proportion of mice at 0–25 m than 150–200 m (two‐way ANOVA, *df* = 3, *F* = 3.48, *p* = .03; Tukey's post hoc tests, *p* = .03) and a significantly higher proportion of males (21.9 ± 16.2% SD) than females (7.5 ± 8.3% SD) overall (two‐way ANOVA, *df* = 1, *F* = 13.05, *p* = .001; Figure 2). Based on a small number of marked and recaptured mice (*n* = 8), we deduced that individuals were able to travel across the trapping grid size easily during our trapping time, with the maximum travel up to approximately 125 m inland in one night. There was no significant change in adult male (*n* = 35; Kruskal–Wallis test, *χ^2^* = 2.85, *df* = 2, *p* = .24) or female (*n* = 19; Kruskal–Wallis test, *χ^2^* = 2.18, *df* = 3, *p* = .54) body mass along distance intervals from the beach at which they were captured.

**FIGURE 3:**
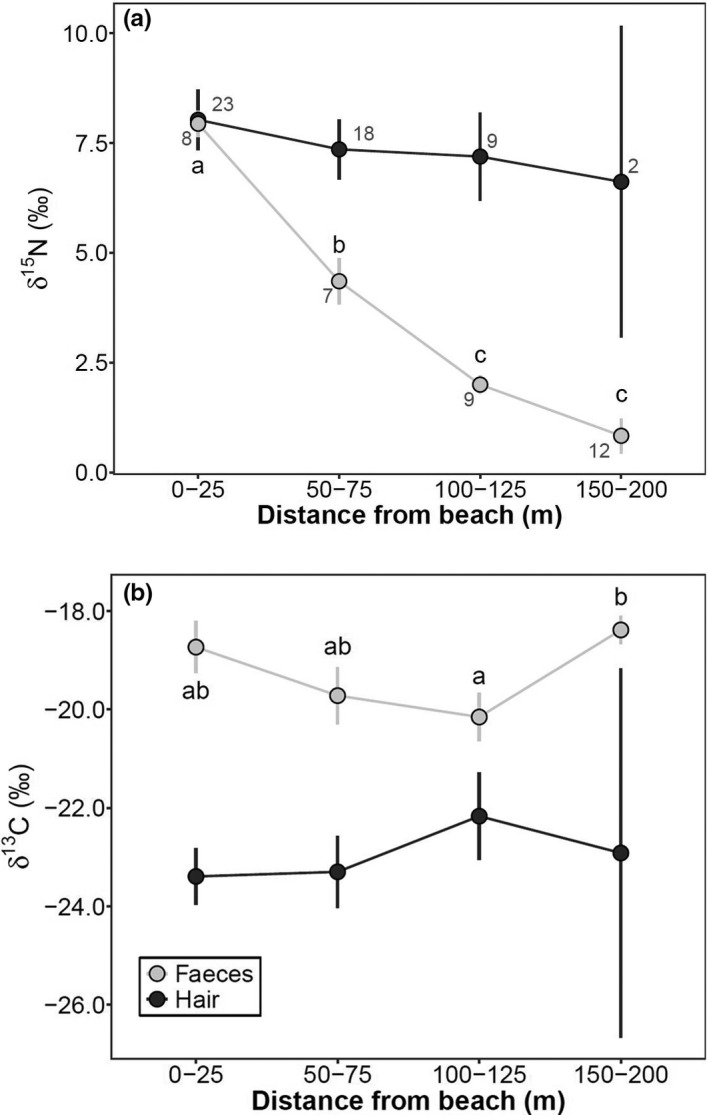
Average (± 1 *SE*) stable isotope signatures of (a) *δ* ^15^N and (b) lipid‐corrected *δ* ^13^C in mouse feces (light gray) and uncorrected *δ* ^13^C in hair (dark gray) from the beach into the forest. Values in a series with different letters are significantly different (Tukey's post hoc tests, *p* < .05); no letters (or the same letters) in a series indicate no statistical significance among distance intervals. No trophic fractionation corrections were applied. Sample sizes are given for *δ* ^15^N and apply to *δ* ^13^C. Fecal samples were collected at Gosling Island and hair samples from all five sites

Mouse fecal *δ* ^15^N stable isotope signatures, which provide a snapshot of diet composition, declined with distance to shore and were significantly different among distance intervals (one‐way ANOVA, *df* = 3, *F* = 70.92, *p* < .001; Figure 3a). Fecal *δ* ^13^C also differed significantly (one‐way ANOVA, *df* = 3, *F* = 3.65, *p* = .02), but only between the 100–125 m and 150–200 m groups (Tukey's post hoc test, *p* = .03; Figure 3b). However, stable isotope signatures in mouse hair, which provide longer‐term, time‐averaged diet over several months, showed no significant differences in either *δ* ^15^N (one‐way ANOVA, *df* = 3, *F* = 0.29, *p* = .83) or *δ* ^13^C signatures (one‐way ANOVA, *df* = 3, *F* = 0.40, *p* = .75) among distance intervals (Figure 3a and b).

**FIGURE 4:**
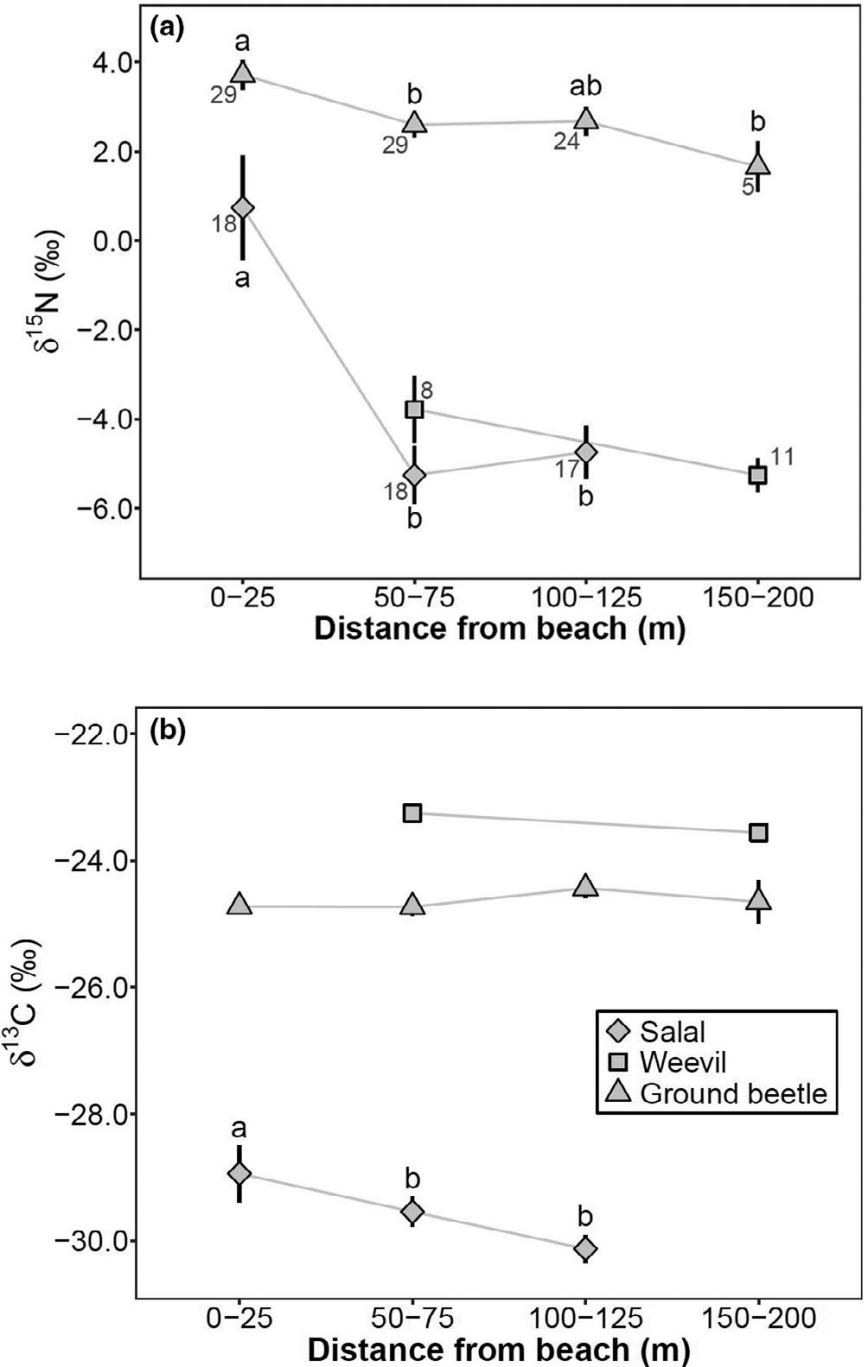
Raw average (± 1 *SE*) stable isotope signatures of (a) *δ* ^15^N and (b) lipid‐corrected *δ* ^13^C in carnivorous ground beetles (triangles) and herbivorous weevils (squares), and uncorrected *δ* ^13^C in salal berries (diamonds) from the beach into the forest. Weevil samples were pooled across 0–75 m and 100–200 m. Values in a series with different letters are significantly different (Tukey's post hoc tests, *p* < .05); no letters (or the same letters) in a series indicate no statistical significance among distance intervals. Sample sizes are given for *δ* ^15^N and apply to *δ* ^13^C. Arthropod samples collected at all sites except Little Grief, plant samples collected at North Beach, South Goose, and Gosling Island

Contrary to mouse captures, we did not find a significant decline in forest arthropod biomass moving inland (one‐way ANOVA, *df* = 3, *F* = 0.56, *p* = .64). Three focal food source groups (salal berries representing vegetative foods, herbivorous weevils, and carnivorous ground beetles) exhibited some patterns in marine enrichment near shorelines (Figure 4a and b). The *δ* ^15^N signatures of ground beetles (Kruskal–Wallis tests, *χ^2^* = 11.55, *df* = 3, *p* = .01) and salal berries (*χ^2^* = 19.43, *df* = 2, *p* < .001) declined significantly moving inland, while *δ* ^15^N in weevils did not (*χ^2^* = 3.01, *df* = 1, *p* = .08). There was no significant change in *δ* ^13^C signatures for ground beetles (*χ^2^* = 1.39, *df* = 2, *p* = .71) or weevils (*χ^2^* = 0.83, *df* = 1, *p* = .36) moving inland, but salal exhibited significant depletion in *δ* ^13^C moving inland (*χ^2^* = 6.97, *df* = 2, *p* = .03).

### 3.2 Variation in diet within populations

**FIGURE 5:**
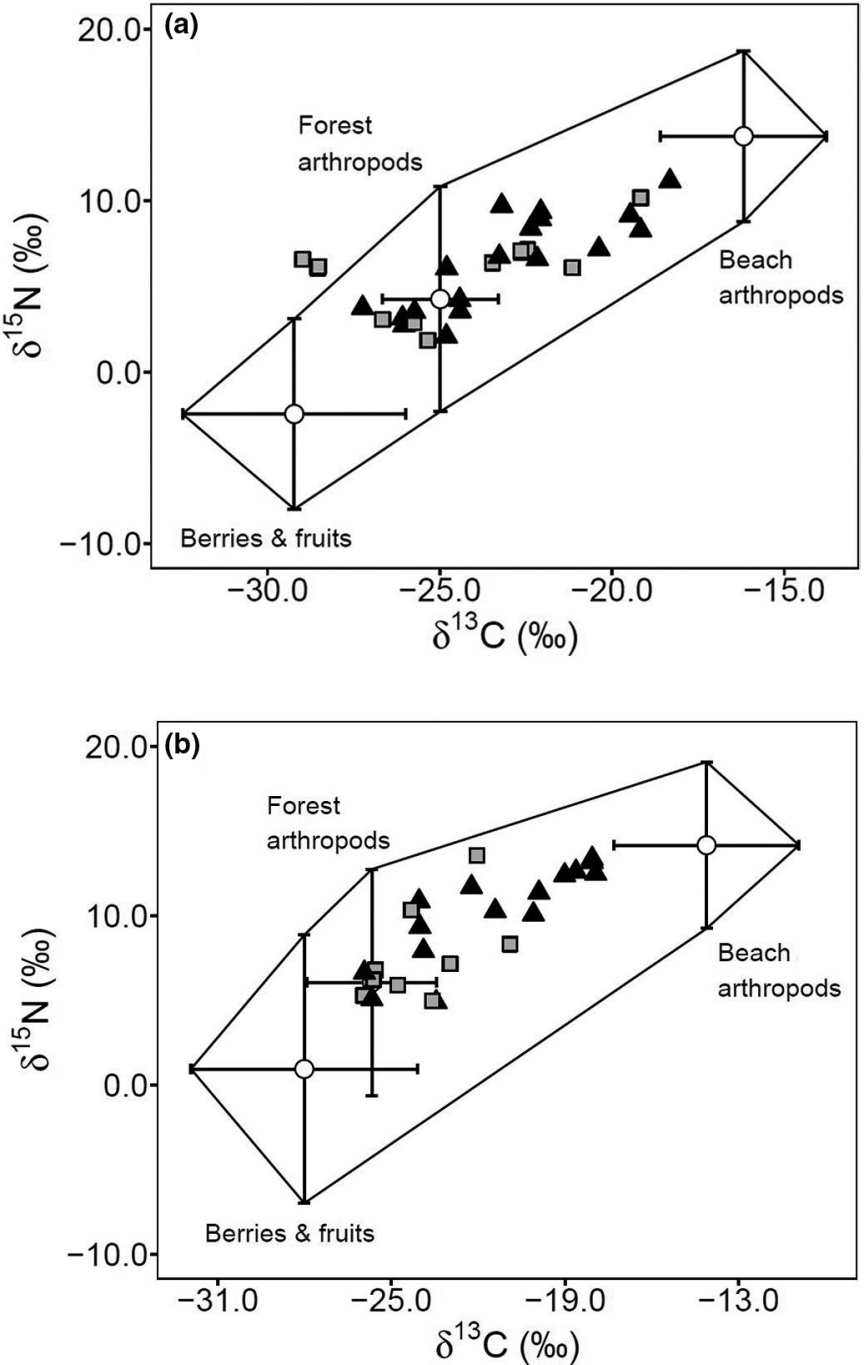
Isotopic mixing model polygons from (a) Calvert Island and (b) Goose Archipelago representing food sources used in region‐specific MixSIAR diet models. Food values (circles) are mean ± *SD* with overlaid individual consumer signatures separated by sex (males = dark triangles, females = light squares)

Individual mice varied substantially in dietary composition. Isotopic mixing models indicated that the proportion of beach arthropods (herbivores and carnivores) in individual diets ranged from 4.9 to 73%. Median values from regional MixSIAR models indicated that approximately one‐third of mouse diets are composed of beach arthropods, with mice on Calvert Island and the Goose Archipelago consuming similar amounts (median = 37.0% and 35.4%, respectively; Table 1 and Figure 5).

**FIGURE A1:**
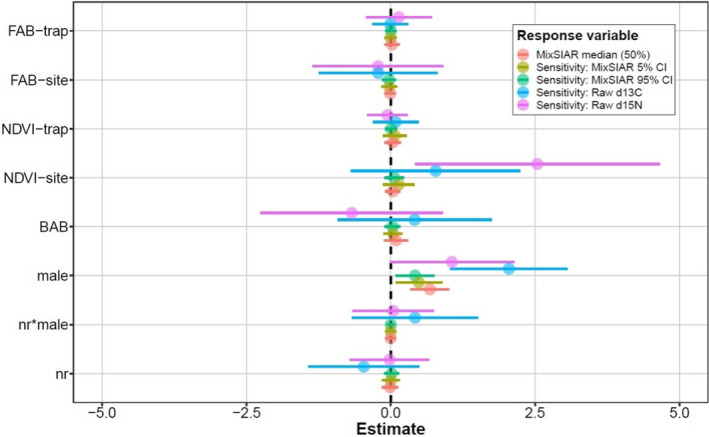
Parameter estimates (± 1 SE) for predictor variables modeling the median (50%) posterior distribution values of proportion of beach arthropods in individual diets from MixSIAR output (red) and four sensitivity modeling exercises. Sensitivity analysis models used the following response variables: the 5% (olive) and 95% (green) posterior distribution values of proportion of beach arthropods in individual diets from MixSIAR output; raw *δ* ^13^C values (blue); and raw *δ* ^15^N (pink) values. Variable codes are as follows: beach arthropod biomass (“BAB”), trap‐level forest arthropod biomass (“FAB‐*trap”*), site‐level forest arthropod biomass (“FAB‐*site”*), trap‐level NDVI (“NDVI‐*trap”*), site‐level Normalized Differentiated Vegetation Index (“NDVI‐*site”*), male mice (“male”), non‐reproductive mice (“nr”), and interactions between non‐reproductive male mice (“male*nr”). Continuous predictors (biomasses and NDVI) were centered and scaled by subtracting the mean and dividing by 2 times the standard deviation

The top model predicting the proportion of beach arthropods in diet contained only mouse sex as a predictor (*w* = 0.19; Table 2), which had 1.5 times the model weight as the following model including mouse sex and beach arthropod biomass (*w* = 0.11). The remaining models had relatively equal weights (Table 2). Full model‐averaged parameter estimates indicated weak relationships between the median proportion of beach arthropods consumed and all predictor variables, with the exception of sex (male mice), which did not overlap zero (Appendix Tables A7, A8 and A7, A8; Appendix Figure A1). Male mice from Calvert Island and the Goose Archipelago consumed more beach arthropods (31.7 ± 21.4% and 44.4 ± 26.3%, respectively) than females from Calvert and Goose (19.9 ± 8.7% and 18.1 ± 13.5%, respectively). Summary statistics for variables included in mixed‐effects models are given in Appendix Table A9.

**TABLE 2:** Top models (95% cumulative GLMM weight) explaining variation in the proportion of beach arthropods consumed by individual mice (MixSIAR median value)

Model	Fixed effects	*L*	*df*	AICc	ΔAICc	*w_i_*
7	Sex	15.66	4	−22.30	0.00	0.190
38	BAB + sex	16.37	5	−21.17	1.13	0.108
21	NDVI‐*site* + sex	16.08	5	−20.59	1.71	0.081
12	NDVI‐*trap* + sex	16.04	5	−20.51	1.79	0.078
27	FAB‐*trap* + sex	15.72	5	−19.87	2.43	0.056
34	FAB‐*site* + sex	15.72	5	−19.85	2.44	0.056
42	reproductive status + sex	15.67	5	−19.75	2.54	0.053
32	FAB‐*trap* + BAB + sex	16.89	6	−19.50	2.80	0.047
25	NDVI‐*site* + BAB + sex	16.56	6	−18.84	3.45	0.034
17	NDVI‐*trap* + BAB + sex	16.52	6	−18.77	3.53	0.033
39	BAB + sex + reproductive status	16.42	6	−18.57	3.72	0.030
24	NDVI‐*site* + sex + reproductive status	16.16	6	−18.05	4.25	0.023
19	NDVI‐*trap* + sex + reproductive status	16.06	6	−17.85	4.45	0.021
15	NDVI‐*trap* + FAB‐*site* + sex	16.04	6	−17.82	4.48	0.020
1	(null)	12.15	3	−17.69	4.60	0.019
37	FAB‐*site* + sex + reproductive status	15.74	6	−17.20	5.10	0.015
30	FAB‐*trap* + sex + reproductive status	15.73	6	−17.19	5.11	0.015
9	sex*reproductive status	15.67	6	−17.07	5.23	0.014
2	BAB	12.94	4	−16.85	5.45	0.012
6	NDVI‐*site*	12.75	4	−16.47	5.82	0.010
4	FAB‐*site*	12.42	4	−15.81	6.49	0.007
40	BAB + sex*reproductive status	16.43	7	−15.74	6.55	0.007
5	NDVI‐*trap*	12.37	4	−15.72	6.57	0.007
8	reproductive status	12.23	4	−15.42	6.87	0.006
3	FAB‐*trap*	12.18	4	−15.34	6.95	0.006

Note

Variable codes are as follows: mouse sex (“sex”), site‐level Normalized Differentiated Vegetation Index (“NDVI‐*site*”), trap‐level NDVI (“NDVI‐*trap*”), site‐level forest arthropod biomass (“FAB‐*site*”), trap‐level forest arthropod biomass (“FAB‐*trap*”), beach arthropod biomass (“BAB”) and mouse reproductive status (“reproductive status”). Continuous predictors were centered and scaled by subtracting the mean and dividing by 2 times the standard deviation. Log‐likelihood (“*L*”), degrees of freedom (“*df*”), Akaike's information criterion (corrected for small sample size, “AICc”), ΔAICc, and AIC weight (“*w_i_*”) are given. Model number corresponds to candidate models in Appendix Table A6 and parameter estimates in Appendix Tables A7, A8 and A7, A8.

These patterns were robust to different analytical approaches. Results from the 5% and 95% posterior distribution dietary estimates were relatively consistent with the median (50%) posterior distribution estimates. The top model contained only “sex,” where males had a higher proportion of beach arthropods in their diets. The importance of other variables (environmental or reproductive, Appendix Table A5) were more variable depending on the response variable, but ultimately had little effect on the proportion of beach arthropods in individual diets as all model‐averaged parameter estimates overlapped zero (Appendix Figure A1). The Gaussian‐distributed LMMs run with raw *δ* ^13^C and *δ* ^15^N values also showed a similar pattern. Male mice showed more enriched *δ* ^13^C and *δ* ^15^N signatures than female mice, with sex parameter estimates differing from zero for *δ* ^13^C models and nearly so for *δ* ^15^N models (1.06 ± 1.08). Additionally, we detected a positive association of site‐level NDVI in the *δ* ^15^N models (Appendix Figure A1).

## 4 DISCUSSION

We found distinct spatial patterns in habitat use and diet in Keen's mice on coastal islands, which indicated evidence of pronounced marine subsidy. Contrary to our predictions, we found that males—and not reproductive females—were detected more frequently near the beach and consumed more marine‐derived nutrients, although the number of mice captured overall was low. Moreover, additional evidence from isotopic signatures in mice and their foods suggest that subsidies may be incorporated via both direct and indirect pathways.

The spatial patterns of occurrence among consumers at each trophic level, together with individual stable isotope signatures, provide insight into the pathways of marine subsidy. We caught significantly more mice (~50%) within 25 m of the beach, similar to the shore‐biased patterns in capture frequencies of *P*. *maniculatus* recorded by others in the Pacific Northwest (McCabe & McTaggart‐Cowan, 1945, Thomas, 1971, but see Marinelli & Millar, 1989) and on arid islands of southern California (Stapp & Polis, 2003a, 2003b). Mouse feces near shorelines were also significantly enriched in *δ* ^15^N, but not *δ* ^13^C; as a proxy for short‐term dietary “snap‐shots,” this pattern suggests the role of an indirect subsidy pathway. Similar patterns in the *δ* ^15^N and *δ* ^13^C signatures of forest arthropods, the key dietary component of mouse diets (see below), further provide evidence to an indirect subsidy pathway. Indeed, berries and fruits near shorelines were significantly *δ* ^15^N‐enriched relative to inland.

Had a direct subsidy pathway been prominent at the time of sampling, we would expect to observe not only enriched *δ* ^15^N but also *δ* ^13^C signatures in the feces of consumers. This is because marine‐derived organisms are enriched in both isotopes relative to terrestrial sources (Michener & Lajtha, 2007), while indirect consumption would only enrich *δ* ^15^N signatures. For example, elevated *δ* ^15^N but constant *δ* ^13^C of invertebrates feeding on Pacific salmon (*Oncorhynchus spp*.) carcasses also indicated indirect subsidy pathways of terrestrial arthropods through *δ* ^15^N enrichment of vegetation and/or soil, rather than via direct consumption of salmon (Hocking & Reimchen, 2002). It is important to note here that the *δ* ^13^C enrichment only applies to animal consumers among our samples, given that plant‐based *δ* ^13^C enrichment is typically due to water stress and not marine subsidy, given that plants do not absorb carbon from soils (Dercon et al., 2006; Farquhar & Richards, 1984; Fitzpatrick, 2018; Gehlhausen et al., 2000).

Whereas these patterns provide evidence for the influence of indirect subsidy, additional results from dietary mixing models and recapture data provide a holistic examination of how marine nutrients may permeate through the terrestrial coastal food web to mouse consumers. Based on the collective data presented here, we hypothesize that at the “top” of our focal food web, mice move throughout the forest (where nests would be located) foraging omnivorously. This was confirmed by hair samples, which represent long‐term integrated diet and showed no spatial pattern in either isotope. Moreover, our modest mark–recapture data indicated that mice can travel across most of the trapping grid in a relatively short time. However, mice evidently occupy shorelines more frequently than forest interiors (as suggested by spatial variation in capture frequencies), permitting them to access marine‐subsidized beach‐dwelling arthropods (primarily amphipods, Family Talitridae). In this beachside habitat, they can also consume *δ* ^15^N‐enriched berries and fruits, as well as forest arthropods, resulting in short‐term *δ* ^15^N dietary enrichment and an apparent indirect subsidy pathway. Given a long‐term “balanced” diet composed of both forest‐ and marine‐derived foods over space and time (as evident in hair signatures and isotope mixing‐model results), fit is possible that short‐term *δ* ^15^N fecal enrichment is an artifact of all food sources being *δ* ^15^N‐enriched nearshore, while beach‐dwelling arthropods are the only *δ* ^13^C‐enriched food source. This may explain why isotopic mixing models estimated approximately one‐third (35–37%) of individual diets were composed of beach arthropods, while other data lend evidence to an indirect subsidy pathway. Furthermore, if the “beach arthropod” food source was removed, the isotopic dietary polygon in Figure 5 would omit most mouse consumers suggesting an incomplete suite of food sources (Phillips et al., 2014). This estimate of significant direct use of marine foods is consistent with previous observations that have also noted mice foraging on beaches (McCabe & McTaggart‐Cowan, 1945) and amphipod (*Orchestroidea* spp.) exoskeletons in stomach content analyses (Thomas, 1971). Therefore, we suggest that while mice do target marine‐subsidized food sources (beach‐dwelling arthropods), the indirect subsidy pathway to other terrestrial food sources likely still plays an important role in the transfer of marine nutrients to island food webs, of which mice likely act as vectors. Despite a diet composed of ~35% marine foods, which is ~75% higher than the similarly estimated median values of salmon consumption (~20% of diet) in a nearby black bear (*Ursus americanus*) system (Service et al., 2018), consumer traits should be considered when inferring the magnitude of subsidy impact; large‐bodied consumers can likely disperse resource subsides farther, thus having a larger overall effect as a subsidy vector (Kopp & Allen, 2021).

A “balanced” diet despite abundant, readily accessible marine‐subsidized prey is expected when considering the ecological and physiological context of omnivorous consumers. Although coastal mice in particular can obtain most of their protein from allochthonous marine‐derived prey (e.g., amphipods and seabird eggs), their diets still consistently contain a mix of other terrestrial plant and animal matter (Bicknell et al., 2020; Drever et al., 2000; Thomas, 1971). Complete consumption of one food source, especially by an omnivore, is unlikely for two reasons. Individual omnivores select foods in order to maintain an optimal balance of macronutrients (proteins, lipids, and carbohydrates) to support overall health, growth, and reproduction (Coogan et al., 2018; Hew et al., 2016; Raubenheimer & Jones, 2006; Solon‐Biet et al., 2014, 2015; Sørensen et al., 2008). Omnivory is also an adaptive life history trait to carry individuals and populations through fluctuating prey availability (Polis & Strong, 1996); whereas prey‐switching may occur to capitalize on resource pulses, exclusive and sustained consumption of a single food source is unlikely, especially when the subsidy is relatively consistent compared with other systems (e.g., Pacific salmon annual spawning migrations).

### 4.1 Variation among individuals and sex‐specific dietary niches

Whereas we hypothesized that reproductive individuals, particularly females, would have more marine resources in their diets due to the energetic demands of reproduction (Hailey et al., 2001; Polis, 1991; Polis & Strong, 1996; Shine, 1989), our results indicated that males in fact consumed more marine resources, regardless of reproductive status or food availability (including the quantity of beach arthropods).

Although the sex effect was most pronounced, our models using “raw” *δ* ^15^N also revealed a positive relationship between increased forest productivity (indicated by higher NDVI values) and *δ* ^15^N. The latter result lends further evidence to an indirect subsidy pathway, whereby sites with higher forest productivity (presumably due to marine resource subsidies) indirectly enriched mouse diets through elevated *δ* ^15^N.

Isotope mixing models allow for insight into foraging niche variation among individuals, but careful consideration of tissue growth and isotopic turnover is necessary when drawing inference from results. It is unclear as to whether mice undergo discrete, major molts influenced by breeding seasons (Brown, 1963; Collins, 1923; Miller et al., 2008) or continuous, minor molts (Collins, 1919, 1923) mediated by localized resource and climate variability (Tabacaru et al., 2011). Molt timing could influence the dietary window captured in hair samples, especially considering plant and arthropod phenology and their influence on the isotopic composition of mouse hair. Accordingly, we do not make direct comparisons between Calvert Island and Goose Archipelago mouse diets in our analyses. However, isotopic analysis of hair from historical specimens collected over several decades (c. 1930–1950) still exhibited significant enrichment of Goose Archipelago mouse diets (*n* = 11; *δ* ^13^C −18.9 ± 1.06, *δ* ^15^N 11.5 ± 0.89) compared with those from Calvert Island (*n* = 63; *δ* ^13^C −20.4 ± 0.16, *δ* ^15^N 2.6 ± 0.38; [$\bar{\text{x}}$ ± *SD*], K. H. Davidson, unpublished data). Furthermore, the consistent enrichment in plants, arthropods, and mice from the Goose Archipelago aligns with the observation that Goose archipelago receives more marine subsidy (via increased wrack biomass) than Calvert Island (Wickham et al., 2020).

Divergence in dietary niche between sexes may represent different trade‐offs faced by males and females within a population. Males may monopolize a high‐quality resource by excluding or out‐competing females, and likewise, females with dependent offspring may avoid aggressive males (e.g., Grizzly bears, *Ursus arctos*, Ben‐David et al., 2004, Rode et al., 2006, Adams et al., 2017). In mice (*Peromyscus* spp. and *Apodemus* sp.), dominant reproductive males will hold territories in higher‐quality edge habitats (Wolf & Batzli, 2002) and maintain larger home ranges than females (Attuquayefio et al., 1986; Blair, 1942; Wolff, 1985), presumably to increase access to food resources (Shine, 1989) or reproductive females (Ims, 1987; Ostfeld, 1985; Wolff, 1985). Therefore, males with larger home ranges may simply encounter beach habitats more frequently than females (with smaller home ranges in forest interiors). Consequently, males may have to trade‐off high‐quality forage with predation risk, as edge habitats are frequently used by mammalian and avian predators and may represent risky habitat (Wolf & Batzli, 2002, 2004). On the small, exposed islands of the BC Central Coast, potential predators such as mink (*Neovison vison*) and wolves (*Canis lupus*) frequently use beaches to access marine resources, but in general, small islands typically support lower abundance and variety of predators than mainlands or very large islands (Adler & Levins, 1994; Gliwicz, 1980; Sullivan, 1977), which is consistent in our study area (Davidson, 2018). In our system, female mice may be more risk‐averse; foraging in complex understory likely provides security against predation risk (Anderson et al., 2003; Anderson & Meikle, 2006), as well as access to den sites (Gosselink et al., 2003). A different context of predation risk might explain why our results differed from Marinelli and Millar (Marinelli & Millar, 1989), who found a higher proportion of pregnant female mice (*Peromyscus maniculatus*) near shorelines compared with temperate forest interiors in the Pacific Northwest.

In addition to external pressures such as predation or habitat choice, individual consumers must balance sex‐specific physiological requirements depending on life stage and food availability. In laboratory studies, mice regulate protein intake more strongly than carbohydrates (Sørensen et al., 2008), but this varies between males and females (Hew et al., 2016; Solon‐Biet et al., 2015). Female reproduction is maximized on either high or low protein diets depending on the reproductive trait of study, while male reproduction is maximized on a balanced protein‐to‐carbohydrate diet (Solon‐Biet et al., 2015). As amphipods (Family Talitridae) represented most of the “beach arthropods” food source and are high in protein, and low in carbohydrate and fat (F. Gammaridae and F. Caprellidae; Baeza‐Rojano et al., 2014), it is possible that the fine‐scale reproductive stage and traits of individual female mice (e.g., pre‐ or post‐pregnancy or estrous cycle stage) may determine whether proteins or carbohydrates are more important, and thus result in more variable foraging decisions compared with male mice.

## 5 CONCLUSIONS

If our examination of Keen's mice on the BC Central Coast is representative of coastal populations elsewhere, then marine subsidies can likely generate intrapopulation variation in both dietary and spatial niche in other omnivores. Sex‐based partitioning of diet and habitat use may have broader implications. Different spatial and temporal behavior between sexes, for example, might influence the patterns in which nutrients are transferred from the ocean to the land, or the population dynamics associated with marine subsidies, especially for omnivores balancing a variety of food sources.

As Leroux and Loreau (2008) identified, coastal ecotones may be difficult to characterize in terms of the allochthonous resource spectrum. Whereas we have not specifically quantified the subsidy–consumer linkage strengths of our study system, we suspect they are likely weaker than those in other coastal island systems where in situ resources are more limited (e.g., Polis & Hurd, 1995; Stapp & Polis, 2003a). Quantifying these food web linkage strengths across coastal systems would further our understanding of resource subsidies in coastal systems. Future studies could also integrate considerations of fine‐scale individual macronutrient balance with experimental manipulations of wrack. Such approaches could assess long‐term subsidy impacts, which is an under‐examined area (Gratton & Denno, 2003; Spiller et al., 2010), and help to understand the mechanisms involved in the flow and mediation of marine nutrient subsidies onto islands.

## CONFLICT OF INTEREST

The authors listed do not declare any conflict of interest.

## AUTHOR CONTRIBUTIONS

**Katie H. Davidson:** Conceptualization (equal); Data curation (equal); Formal analysis (lead); Funding acquisition (supporting); Investigation (lead); Methodology (lead); Project administration (equal); Validation (lead); Visualization (equal); Writing‐original draft (lead); Writing‐review & editing (lead). **Brian M. Starzomski:** Conceptualization (equal); Formal analysis (supporting); Funding acquisition (lead); Investigation (supporting); Methodology (supporting); Resources (equal); Supervision (supporting); Writing‐review & editing (equal). **Rana El‐Sabaawi:** Conceptualization (supporting); Formal analysis (supporting); Investigation (supporting); Methodology (supporting); Supervision (supporting); Writing‐review & editing (equal). **Morgan D. Hocking:** Conceptualization (supporting); Formal analysis (supporting); Investigation (supporting); Methodology (supporting); Supervision (supporting); Writing‐review & editing (equal). **John D. Reynolds:** Conceptualization (equal); Formal analysis (supporting); Funding acquisition (lead); Investigation (supporting); Methodology (supporting); Resources (equal); Supervision (supporting); Writing‐review & editing (equal). **Sara B. Wickham:** Conceptualization (equal); Data curation (supporting); Formal analysis (supporting); Investigation (supporting); Methodology (supporting); Resources (equal); Visualization (supporting); Writing‐review & editing (equal). **Chris T. Darimont:** Conceptualization (equal); Data curation (supporting); Formal analysis (supporting); Funding acquisition (lead); Investigation (supporting); Methodology (supporting); Project administration (equal); Resources (lead); Supervision (lead); Visualization (supporting); Writing‐original draft (equal); Writing‐review & editing (equal).

### OPEN RESEARCH BADGES

This article has earned an Open Data, for making publicly available the digitally‐shareable data necessary to reproduce the reported results. The data is available at https://catalogue.hakai.org/dataset/ca‐cioos_82c07005‐9313‐436c‐9239‐7be3f5907be2.

## Data Availability

All data used in analyses here are stored in the Hakai Institute's data repository, which is open source (https://catalogue.hakai.org/dataset/ca‐cioos_82c07005‐9313‐436c‐9239‐7be3f5907be2). All code for analyses is available on K. H. Davidson's github repository (https://github.com/khdavidson/davidsonetal‐keensmouse.git).

## 1 Methods

### 1.1 Ethics and data accessibility statement

Small mammal trapping was approved under University of Victoria Animal Use Permit #2016‐012 in accordance with Canadian Council on Animal Care guidelines and followed the university's Standard Operating Protocols (SOP) AC2007 and AC2023. Trapping and survey methods complied with the British Columbia Resources Information Standards Committee (BC RISC) Inventory Methods for Small Mammals (Ministry of Environment, Lands and Parks Resources Inventory Branch Report No. 31, Version 2.0, 1998) and the American Society of Mammalogists Guidelines (Sikes, 2016). All field researchers completed animal handling training sessions through the University of Victoria Animal Care Services. Field work was conducted out of the Hakai Institute on Calvert Island, BC, within the Hakai Lúxvbálís Conservancy area under a long‐term operation BC Parks Use Permit No. 107190. Field work was conducted within the traditional territories of the Haíɫzaqv (Heiltsuk) and Wuikinuxv First Nations after receiving their permission via a research protocol with the Hakai Institute. Traditional names of animals and plants are included in Appendix tables (where available) and were sourced from the online’Uik̓ala language dictionary (First Voices,’Wuik̓ala/’Uik̓ala), Biodiversity of the Central Coast (www.centralcoastbiodiversity.org), and Compton (1993). All data used in analyses here are stored in the Hakai Institute's data repository, which is open source (https://catalogue.hakai.org/dataset/ca‐cioos_82c07005‐9313‐436c‐9239‐7be3f5907be2). All code for analyses is available on K. H. Davidson's github repository (https://github.com/khdavidson/davidsonetal‐keensmouse.git).

### 1.2 Mouse biometrics

Sex was determined using the anogenital distance, or reproductive traits when visible. Males were considered reproductive when testes were scrotal and enlarged; females were considered reproductive if they were in later stages of estrus (proestrus and estrus) or if there was evidence of lactation (worn fur around papillae), which indicated a recent litter and reproductive activity. Adults and sub‐adults were differentiated based on size and colouration, with adults exhibiting redder pelage, often with a distinct black dorsal band, compared to gray‐coloured juveniles (Collins, 1923; McCabe & McTaggart‐Cowan, 1945). Mice were weighed using PESOLA^®^ spring scales and rear right foot length measured using Blindman's^®^ fractional electronic calipers.

### 1.3 Trap designs

Mouse live traps were baited with peanut butter, and supplemented with carrot for hydration and mealworms for additional protein (particularly to prevent mortality of shrew [*Sorex* spp.] by‐catch). To minimize potential heat loss, we also provided a synthetic batting for ‘warm‐when‐wet’ nesting material, and rain covers made of waxed Tetra‐Pak^®^. We did not conduct trapping on nights when heavy rain was anticipated to reduce risk to mice and non‐target shrews (*Sorex* spp.), the latter of which are extremely sensitive to heat loss. Traps were set at approximately 1800 h and collections begun at ~0600 h the following morning. Captured mice were transferred from traps to perforated plastic bags (large Ziploc^®^ Produce Bags) for processing. To minimize risk of disease or parasite transmission, bags were disposed of after handling individual mice, and all tools were sterilized with 10% bleach followed by a water rinse and 95% ethanol between individuals. Mouse track plates (for faecal sample collection) were housed in PVC/ABS pipe and followed the design of Nams and Gillis (2003). Arthropod pitfall traps contained a 3:1 solution of propylene:glycol and fresh water with a small amount (~1 ml) of detergent (Tritin X) to break the surface tension.

### 1.4 Invertebrate sample processing

We estimated beach and forest arthropod biomass by randomly selecting up to 30 individuals from the most common taxa (95% of identified counted taxa sampled, Table S1). Individuals were dried for 24–48 h (depending on body size and moisture content) at 60°C and weighed on a microbalance (model as above). Based on these weights, an average weight per individual for each taxon was obtained and used to convert the remaining count data to biomass data (Table 2.6). Some species exhibiting major body size variation within taxa were binned into two size classes representing small and large morphs and 30 individuals from each size class were sampled: spiders were binned into ≤3mm and >3 mm in length, and Staphylinid beetles and Talitrid amphipods into ≤5 mm and >5 mm in length. Aerial insects (e.g., adult forms of flies, bees and wasps) were not included in our calculations as they are unlikely to be consumed by rodents, nor were unidentifiable and rare individuals. We summed the total arthropod dry biomass in each trap, correcting for varied trap effort (1–6 nights depending on location and transect), and averaged values across each distance interval (± standard error). These data were analyzed along a spatial gradient from the beach back ~200m into the forest to determine if there was evidence of subsidy in biomass patterns.

### 1.5 Normalized Differentiated Vegetation Index (NDVI)

NDVI data were obtained from the Worldview2 sensor at 2‐m resolution for GOS and GS‐S 14‐August‐2014, for NB 4‐August‐2014, and for GF and IP 4‐June‐2015. Although these dates vary, there was no significant difference in NDVI values between image month‐year combinations (Kruskal–Wallis Test, *χ^2^* = 0.31, *df* = 1, *p* = .58). We used this as a proxy for forest primary productivity within predictive models to represent available plant foods at each site, as ground‐based estimates of plant food biomass were not available. NDVI has been used predict fruit and seed yields (Camarero et al., 2010; Li et al., 2010), diets and population dynamics in large herbivores (Cerling et al., 2006; Creech et al., 2016; Pettorelli et al., 2011), and in some cases omnivores and carnivores (Andreo et al., 2009; Andreo et al., 2009; Cerling et al., 2006; Creech et al., 2016; Pettorelli et al., 2011). Furthermore, its fine‐scale resolution and continuous nature makes it useful for modelling (Pettorelli et al., 2011). However, we acknowledge that NDVI is representative of the upper canopy and not the shrub layer, where many fruits are produced (e.g., salal or huckleberry), and may not provide an instantaneous estimation of food availability (Creech et al., 2016).

### 1.6 Stable isotope analysis

Samples stored in ethanol (plants, arthropods and faecal matter) were rinsed three times in deionized water, dried at 60°C for 24–48 h (large‐bodied arthropods, e.g., slugs and ground beetles, were dried longer), and ground in a ball mill grinder (Retsch Mixer Mill MM200). For berries and fruits, we homogenized 3–10 individuals per sample, depending on size. The number of arthropods per sample varied by body size, ranging from one individual per sample for large arthropods (e.g., Carabid ground beetles) to 60–80 individuals per sample (e.g., Collembola). All faecal matter collected from a track plate was pooled into one sample. We rinsed and cleaned mouse hair samples in 2:1 chloroform‐methanol to remove surface oils and dirt, and dried samples overnight in a fume hood.

Samples were encapsulated in tin capsules at average sample weights of 1.23 ± 0.13 mg, 3.44 ± 0.53 mg, 1.21 ± 0.09 mg, and 1.27 ± 0.06 mg for arthropod, plant, hair, and faecal tissue, respectively, using a microbalance (Mettler‐Toledo MX5, accurate to 0.001 mg). Samples were sent to the University of California Davis Stable Isotope Facility (see http://stableisotopefacility.ucdavis.edu/13cand15n.html for details). The long‐term standard deviation for the lab is 0.2 permil (‰) for ^13^C and 0.3 permil (‰) for ^15^N. The final delta values, δ^13^C (e.g., Equation 1) and δ^15^N, are expressed relative to international standards V‐PDB (Vienna PeeDee Belemnite) and air for carbon and nitrogen, respectively.

$$\delta^{\mathrm{xx}}E=\frac{R_{\text{sample}}‐R_{\text{standard}}}{R_{\text{standard}}}\cdot 1000‰$$

where *E* is the element of interest, *^xx^* is the mass of the heavier isotope in the ratio, and *R* is the ratio of the heavy to light isotope within a sample or standard material.

**TABLE A1:** Invertebrate taxonomic groups and their source (F = forest, B = beach) and capture regions (CV = Calvert Island, GS = Goose Archipelago). The number of individuals used to calculate mean body mass (± *SD* d/w) per group were eventually used to calculate invertebrate biomass per region for Generalize Linear Mixed‐effects Models (GLMMs). For highly abundant groups, a subsample (*n* = 30) was used to calculate average individual biomass. Haíɫzaqv (Heiltsuk, ‘H’) and’Uik̓ala (Wuikinuxv, ‘U’) names are given where available

Taxonomic group	H / U name	Source	Region	Individuals sampled	Individual dry weight (mg)
Amphipods (≤5 mm, *Traskorchestia traskiana*, *Megalorchestia columbiana*)	—	B	GS, CV	30	0.29 ± 0.20
Amphipods (>5 mm, *Traskorchestia traskiana*, *Megalorchestia columbiana*)	—	B	GS, CV	30	6.65 ± 3.58
Ants (F. Formicidae)	K̓ázálác̓i / W̓isw̓əluy̓uqvs	F, B	GS, CV	23	0.98 ± 0.48
Camel cricket (*Pristoceuthophilus celatus*)	—	F	CV	10	7.09 ± 6.09
Carabid ground beetles (*Bembidion* sp.)	—	B	CV	26	2.59 ± 0.50
Carabid ground beetles (*Scaphinotus angusticollis*, *Pterostichus lama*, *Zactous matthewsii*, *Cychrus tuberculatus*)	—	F	CV, GS	33	73.37 ± 50.49
Centipede (*Scolopocryptops spinicaudus*)	—	F	CV, GS	9 (6)	15.06 ± 25.45^a^ (1.01 ± 1.05)
Isopods (O. Isopoda)	—	F, B	CV, GS	16	1.50 ± 1.00
Mites (F. Trombidiidae, other unidentifiable Acariformes)	—	F, B	CV, GS	30	0.39 ± 0.29
Pictured rove beetle (*Thinopinus pictus*)	—	B	CV, GS	30 (28)	4.49 ± 13.82^a^ (0.88 ± 1.08)
Rove beetles (≤ 5 mm, F. Staphylinidae)	—	B	CV, GS	30	0.25 ± 0.16
Rove beetles (> 5 mm, F. Staphylinidae)	—	B	CV, GS	32	5.90 ± 2.99
Snails (*Ancotrema sportella*, *Vespericola columbianus*)	— / Q̓vàdas	F	CV, GS	30	51.64 ± 42.25^b^
Spiders (≤ 3 mm, mostly F. Linyphiidae)	Húmáx̌a / Haùmax̌a	F, B	CV, GS	30	0.29 ± 0.52
Spiders (> 3 mm, mostly F. Lycosidae; húmáx̌a, haùmax̌a)	Húmáx̌a / Haùmax̌a	F, B	CV, GS	30	8.37 ± 9.97
Springtails (O. Entomobryomorpha, Poduromorpha, Symphypleona)	—	F, B	CV, GS	29	0.074 ± 0.045
Weevil (*Steremnius carinatus*)	—	F, B	CV, GS	30	13.68 ± 3.19

^a^ Includes some exceptionally large individuals; values in parentheses omit these outliers. All individuals were included in biomass calculations.

^b^ Shells removed.

**TABLE A2:** Wilcoxon (Mann–Whitney) test results comparing regional (GS and CV) δ^13^C and δ^15^N stable isotope signatures of mice and food items. *W*‐statistic, *p*‐values, average (± SE) δ^13^C and δ^15^N values, and sample sizes (in parentheses) are given. No trophic fractionation was applied to food items. Bolded values indicate the more enriched region. Asterisks identify significant results

Group	δ^13^C				δ^15^N			
	CV	GS	*W*	*p*	CV	GS	*W*	*p*
Vegetation^b^	−31.2 ± 0.28 (19)	**−30.54 ± 0.20** (81)	594.5	.13	−5.76 ± 0.52 (19)	**−2.66 ± 0.44** (81)	360	<.001^a^
Forest arthropods	**−26.0 ± 0.68 (76)**	−26.7 ± 2.2 (68)	3229	.01^a^	0.95 ± 3.3 (76)	**2.76 ± 3.4 (68)**	1635	<.001^a^
Beach arthropods	−17.2 ± 1.41 (47)	**−15.1 ± 2.2 (54)**	529.5	<.001^a^	10.5 ± 1.68 (47)	**10.9 ± 1.62 (54)**	1048	.13
Mouse hair (*P*. *keeni*)	−23.58 ± 0.51 (31)	**−22.56 ± 0.54** (25)	308	.26	6.37 ± 0.45 (31)	**8.88 ± 0.64** (25)	220	.009^a^

^a^ Significant result (*p* < .05)

^b^ Comparison only includes salal (*Gaultheria shallon*) and huckleberry (*Vaccinium parvifolium*), the two species collected in both regions, but MixSIAR models include more region‐specific berry and fruit species (see Tables S2 and S3).

**TABLE A3:** Species composing each food group used in stable isotope analysis and MixSIAR modelling for Calvert Island. Haíɫzaqv (Heiltsuk, ‘H’) and’Uik̓ala (Wuikinuxv, ‘U’) names are given where available

Region	Food group	Species	H / U Name	Tissue	Number of samples	Individuals per sample
Calvert Island	Vegetation	Red huckleberry (*Vaccinium parvifolium*)	Ĝvádṃ / Ĝvàdəm	Berry	16	10–12
		Salal (*Gaultheria shallon*)	Nk̓vɫ / Nk̓vt	Berry	3	10
	Forest arthropods	Weevil (*Steremnius carinatus*)	—	Whole body	11	5–7
		Springtails (O. Entomobryomorpha, Poduromorpha, Symphypleona)	—	Whole body	4	>100
		Isopod (O. Isopoda)	—	Whole body	1	5–10
		Centipede (*Scolopocryptops spinicaudus*)	—	Whole body	1	5
		Carabid ground beetles (*Scaphinotus angusticollis*, *Pterostichus lama*, *Zactous matthewsii*, *Cychrus tuberculatus*)	—	Whole body	42	1–10
		Spiders (F. Linyphiidae, Lycosidae)	Húmáx̌a / Haùmax̌a	Whole body	6	~30^a^
	Beach arthropods	Amphipods (*Traskorchestia traskiana*, *Megalorchestia columbiana*)	—	Whole body	30	10
		Spiders (F. Linyphiidae, Lycosidae)	Húmáx̌a / Haùmax̌a	Whole body	3	~30^a^
		Rove beetle (F. Staphylinidae)	—	Whole body	8	5
		Carabid ground beetle (*Bembidion* sp.)	—	Whole body	4	10
		Rove beetle (*Thinopinus pictus*)	—	Whole body	2	15–30
	Consumer	Mice (*Peromyscus keeni*)	H̓skáníqs / A̓skaniqs	Hair	30	1

^a^ Varied depending on body size.

**TABLE A4:** Species composing each food group used in stable isotope analysis and MixSIAR modelling for the Goose Archipelago. Haíɫzaqv (Heiltsuk, ‘H’) and’Uik̓ala (Wuikinuxv, ‘U’) names are given where available

Region	Food group	Species	H / U Name	Tissue	Number of samples	Individuals per sample
Goose Archipelago	Vegetation	Red huckleberry (*Vaccinium parvifolium*)	Ĝvádṃ / Ĝvàdəm	Berry	30	10–12
		Salal (*Gaultheria shallon*)	Nk̓vɫ / Nk̓vt	Berry	51	10
		Pacific crab apple (*Malus fusca*)	K̓vṃ́tkv / Lhə̀nnx̌	Fruit	18	3–5
		Crowberry (*Empetrum nigrum*)	—	Berry	6	10
	Forest arthropods	Weevil (*Steremnius carinatus*)	—	Whole body	8	5–7
		Springtails (O. Entomobryomorpha, Poduromorpha, Symphypleona)	—	Whole body	3	> 100
		Isopod (O. Isopoda)	—	Whole body	1	5–10
		Centipede (*Scolopocryptops spinicaudus*)	—	Whole body	1	5
		Carabid ground beetles (*Scaphinotus angusticollis*, *Pterostichus lama*, *Zactous matthewsii*, *Cychrus tuberculatus*)	—	Whole body	45	1–10
		Spiders (F. Linyphiidae, Lycosidae)	Húmáx̌a / Haùmax̌a	Whole body	6	~30^a^
	Beach arthropods	Amphipods (*Traskorchestia traskiana*, *Megalorchestia columbiana*)	—	Whole body	20	10
		Weevils (*Steremnius carinatus*)	—	Whole body	11	5–7
		Ants (F. Formicidae)	K̓ázálác̓i / —	Whole body	1	20–30
		Spiders (F. Linyphiidae, Lycosidae)	Húmáx̌a / Haùmax̌a	Whole body	9	~30^a^
		Rove beetle (F. Staphylinidae)	—	Whole body	12	5
		Rove beetle (*Thinopinus pictus*)	—	Whole body	1	15–30
	Consumer	Mice (*Peromyscus keeni*)	H̓skáníqs / A̓skaniqs	Hair	24	1

^a^ Varied depending on body size.

**TABLE A5:** Overview of response and predictor variables used models (generalized linear mixed‐effects ‘GLMM’ or linear mixed‐effects ‘LMM’) to examine variation in diet among individual mice (including sensitivity analyses). ‘Description/derivation’ explains the steps taken to arrive at the final variable, while ‘Processing’ explains further processing undertaken (e.g., centered and standardizing) before use

Variable type	Variable category	Variable name	Description/derivation	Processing for models
Response		δ^13^C	Model assessment: Raw δ^13^C of individual mouse hair (LMM)	None
		δ^15^N	Model assessment: Raw δ^15^N of individual mouse hair (LMM)	None
		% beach arthropods in diet	Core analysis: Median probability (50%) from MixSIAR (GLMM) Model assessment: 5% and 95% probability from MixSIAR (GLMM)	None
Predictor	Environmental	beach arthropod biomass	a) Calculated total biomass per trap b) Averaged total biomass per trap at each site	Centered and standardized: $\frac{x‐\bar{x}}{2\sigma }$ Collinearity analysis
		forest arthropod biomass (trap‐level)	a) Identified pitfall trap at location of mouse capture, plus a buffer of 1 adjacent pitfall trap (max 8 traps) b) Calculated total biomass per trap c) Averaged total biomass of these traps	Centered and standardized Collinearity analysis
		forest arthropod biomass (site‐level)	a) Calculated total biomass per trap b) Averaged total biomass per trap at each site	Centered and standardized Collinearity analysis
		NDVI (trap‐level)	a) Assigned an NDVI polygon score to each trap by creating a 5m buffer around the trap and averaging NDVI scores b) Identified NDVI polygon at location of mouse capture, plus a buffer of 1 adjacent NDVI polygons (max 8 polygons) c) Averaged NDVI scores of these trap polygons	Centered and standardized Collinearity analysis
		NDVI (site‐level)	a) Assigned an NDVI polygon score to each trap by creating a 5m buffer around the trap and averaging NDVI scores c) Averaged NDVI polygon scores at each site	Centered and standardized Collinearity analysis
	Individual	Sex	Individual sex: male, female, unknown	Unknowns excluded
		reproductive status	Individual breeding status.	Sub‐adults excluded
	Random effect	Site	A random effect to account for other differences among sites that may influence mouse diet (e.g., interspecific competition).	Little Grief (LG) excluded as environmental variables were unavailable

**TABLE A6:** Candidate model set to assess the effect of ecological and behavioural variables on the proportion of marine foods in individual diets. Fixed effects include the biomass of beach arthropods (‘BAB’), biomass of forest arthropods (‘FAB’), a measure of vegetation productivity (‘NDVI’), and a combined gender‐reproductive status (RG) of individual mice. Italicized words (*site* and *trap*) indicate the spatial scale at which the variable was calculated. Site is a random effect with 4 levels

Model	Fixed effects	Random effects
1	Null	Site
2	BAB	Site
3	FAB‐*site*	Site
4	FAB‐*trap*	Site
5	NDVI‐*site*	Site
6	NDVI‐*trap*	Site
7	Sex	Site
8	Reproductive status	Site
9	Sex * reproductive status	Site
10	NDVI‐*trap* + FAB‐*site*	Site
11	NDVI‐*trap* + BAB	Site
12	NDVI‐*trap* + sex	Site
13	NDVI‐*trap* + reproductive status	Site
14	NDVI‐*trap* + sex * reproductive status	Site
15	NDVI‐*trap* + FAB‐*site* + sex	Site
16	NDVI‐*trap* + FAB‐*site* + reproductive status	Site
17	NDVI‐*trap* + BAB + sex	Site
18	NDVI‐*trap* + BAB + reproductive status	Site
19	NDVI‐*trap* + sex + reproductive status	Site
20	NDVI‐*site* + BAB	Site
21	NDVI‐*site* + sex	Site
22	NDVI‐*site* + reproductive status	Site
23	NDVI‐*site* + sex * reproductive status	Site
24	NDVI‐*site* + sex + reproductive status	Site
25	NDVI‐*site* + BAB + sex	Site
26	NDVI‐*site* + BAB + reproductive status	Site
27	FAB‐*trap* + sex	Site
28	FAB‐*trap* + reproductive status	Site
29	FAB‐*trap* + sex * reproductive status	Site
30	FAB‐*trap* + sex + reproductive status	Site
31	FAB‐*trap* + BAB	Site
32	FAB‐*trap* + BAB + sex	Site
33	FAB‐*trap* + BAB + reproductive status	Site
34	FAB‐*site* + sex	Site
35	FAB‐*site* + reproductive status	Site
36	FAB‐*site* + sex * reproductive status	Site
37	FAB‐*site* + sex + reproductive status	Site
38	BAB + sex	Site
39	BAB + reproductive status	Site
40	BAB + sex * reproductive status	Site
41	BAB + sex + reproductive status	Site
42	Sex + reproductive status	Site

## 2 Results

**TABLE A7:** Parameter estimates for variables in the top models (95% cumulative GLMM weight) predicting variation in the proportion of beach arthropods consumed by individual mice (MixSIAR median probability). Variable codes are: beach arthropod biomass (‘BAB’), trap‐level forest arthropod biomass (‘FAB‐*trap*’), site‐level forest arthropod biomass (‘FAB‐*site*’), trap‐level NDVI (‘NDVI‐*trap*’), site‐level Normalized Differentiated Vegetation Index (‘NDVI‐*site*’), male mice (‘male’), non‐reproductive mice (‘nr’), and interactions between non‐reproductive male mice (‘male*nr’). Standard error is given in parentheses. Bolded estimates do not overlap zero. Model number correspond to candidate models in Appendix Table A6 and model rankings in Table 2

Model	Intercept	BAB^a^	FAB‐*trap* ^a^	FAB‐*site* ^a^	NDVI‐*trap* ^a^	NDVI‐*site* ^a^	Male	nr	male*nr
7	**−1.237 (0.235)**	—	—	—	—	—	**0.734 (0.274)**	—	—
38	**−1.228 (0.222)**	**0.309 (0.253)**	—	—	—	—	**0.718 (0.269)**	—	—
21	**−1.223 (0.223)**	—	—	—	—	0.235 (0.255)	**0.711 (0.271)**	—	—
12	**−1.247 (0.224)**	—	—	—	0.231 (0.266)	—	**0.747 (0.271)**	—	—
27	**−1.259 (0.244)**	—	0.104 (0.297)	—	—	—	**0.761 (0.584)**	—	—
34	**−1.227 (0.240)**	—	—	−0.089 (0.267)	—	—	**0.719 (0.278)**	—	—
42	**−1.220 (0.583)**	—	—	—	—	—	**0.732 (0.275)**	−0.029 (0.274)	—
32	**−1.274 (0.227)**	**0.451 (0.289)**	**0.311 (0.304)**	—	—	—	**0.784 (0.277)**	—	—
25	**−1.222 (0.222)**	0.261 (0.264)	—	—	—	0.163 (0.267)	**0.706 (0.269)**	—	—
17	**−1.239 (0.223)**	0.263 (0.266)	—	—	0.149 (0.278)	—	**0.730 (0.270)**	—	—
39	**−1.177 (0.278)**	**0.323 (0.257)**	—	—	—	—	**0.707 (0.271)**	−0.081 (0.262)	—
24	**−1.154 (0.284)**	—	—	—	—	0.267 (0.267)	**0.695 (0.274)**	−0.106 (0.272)	—
19	**−1.218 (0.277)**	—	—	—	0.236 (0.268)	—	**0.742 (0.273)**	−0.047(0.261)	—
15	**−1.248 (0.228)**	—	—	0.005 (0.285)	0.233 (0.290)	—	**0.748 (0.277)**	—	—
1	**−0.767 (0.141)**	—	—	—	—	—	—	—	—
37	**−1.194 (0.291)**	—	—	−0.104 (0.281)	—	—	**0.712 (0.280)**	−0.055 (0.280)	—
30	**−1.243 (0.294)**	—	0.104 (0.300)	—	—	—	**0.758 (0.285)**	−0.027 (0.276)	—
9	**−1.236 (0.375)**	—	—	—	—	—	**0.754 (0.439)**	−0.003 (0.493)	−0.038 (0.579)
2	**−0.771 (0.140)**	**0.341 (0.265)**	—	—	—	—	—	—	—
6	**−0.769 (0.140)**	—	—	—	—	**0.291 (0.265)**	—	—	—
4	**−0.768 (0.141)**	—	—	−0.201 (0.271)	—	—	—	—	—
40	**−1.201 (0.364)**	**0.323 (0.257)**	—	—	—	—	**0.741 (0.426)**	−0.044 (0.449)	−0.056 (0.548)
5	**−0.768 (0.141)**	—	—	—	0.189 (0.283)	—	—	—	—
8	**−0.706 (0.208)**	—	—	—	—	—	—	−0.109 (0.274)	—
3	**−0.767 (0.141)**	—	−0.074 (0.274)	—	—	—	—	—	—

^a^ Continuous predictors were centered and scaled by subtracting the mean and dividing by 2‐times the standard deviation.

**TABLE A8:** Parameter estimates after model averaging (full‐average) the top GLMMs (95% cumulative weight) predicting variation in the proportion of beach arthropods consumed by individual mice (MixSIAR median estimate). Variable codes are: male mice (‘sex (male)’), beach arthropod biomass (‘BAB’), site‐level Normalized Differentiated Vegetation Index (‘NDVI‐*site*’), trap‐level NDVI (‘NDVI‐*trap*’), site‐level forest arthropod biomass (‘FAB‐*site*’), trap‐level forest arthropod biomass (‘FAB‐*trap*’), non‐reproductive mice (‘reproductive status (non‐reproductive)’), and the interaction between sex and breeding status (‘male*non‐reproductive’). Continuous predictors were centered and scaled by subtracting the mean and dividing by 2‐times the standard deviation. Parameter estimates in bold do not overlap zero and correspond to Appendix Figure A1

Predictor	Estimate	SE	*z*	*p*
Intercept	**−1.198**	**0.269**	4.349	<.001
sex (male)	**0.679**	**0.329**	2.020	.04
BAB	0.093	0.206	0.444	.66
NDVI‐*site*	0.035	0.133	0.262	.79
NDVI‐*trap*	0.036	0.138	0.253	.80
FAB‐*site*	0.023	0.130	0.171	.86
FAB‐*trap*	−0.008	0.093	0.087	.93
reproductive stats (non‐reproductive)	−0.010	0.135	0.072	.94
male*non‐reproductive	−0.000	0.085	0.011	.99

**TABLE A9:** Summary statistics by site for predictor variables included in models to predict variation in the consumption of beach‐dwelling arthropods among individual mice. Biomasses (dry weight) and NDVI are means ± standard deviation. The percentage of reproductive individuals and females are given, with the sample size of individuals in parentheses

Variable	Goose Archipelago		Calvert Island	
	Gosling Island	Goose South	North Beach	Grief Bay
NDVI	0.58 ± 0.01	0.61 ± 0.02	0.54 ± 0.02	0.59 ± 0.01
Forest arthropod biomass (mg/trap)	42.8 ± 45.0	11.5 ± 14.6	45.0 ± 59.3	13.1 ± 13.2
Beach arthropod biomass (mg/trap)	464.4 ± 91.5	388.9 ± 68.6	300.6 ± 31.8	1261.5 ± 321.7
Reproductive individuals*	42% (17)	28.6% (7)	63.6% (11)	33.3% (9)
Female %^*^	42% (17)	28.6% (7)	45.5% (11)	33.3% (9)
